# The utility and cost of routine follow-up procedures in the surveillance of ovarian and primary peritoneal carcinoma: a 16-year institutional review

**DOI:** 10.1038/sj.bjc.6605963

**Published:** 2010-11-02

**Authors:** N B Rettenmaier, C R Rettenmaier, T Wojciechowski, L N Abaid, J V Brown, J P Micha, B H Goldstein

**Affiliations:** 1 Department of Molecular, Cell and Developmental Biology, UCLA College of Letters and Science, Los Angeles, CA, USA; 2 Gynecologic Oncology Associates, 351 Hospital Road, Suite 507, Newport Beach, CA 92663, USA

**Keywords:** ovarian cancer recurrence, CA-125, physical exam, vaginal cytology, imaging, follow-up

## Abstract

**Background:**

The purpose of this study was to evaluate the number of ovarian cancer and primary peritoneal cancer (PPC) progressive disease cases identified via routine follow-up procedures and the corresponding cost throughout a 16-year period at a single medical institution.

**Methods:**

Previously undiagnosed epithelial ovarian (*n*=241), PPC (*n*=23), and concurrent ovarian and uterine (*n*=24) cancer patients were treated and then followed via CA-125, imaging (e.g., CT scan, chest X-ray), physical examination and vaginal cytology.

**Results:**

In the group of 287 patients, there were 151 cases of disease progression. Serial imaging detected the highest number of progressive disease cases (66 initial and 45 confirmatory diagnoses), but the cost was rather high ($13 454 per patient recurrence), whereas CA-125 testing (74 initial and 20 corroborative diagnoses) was the least expensive ($3924) per recurrent diagnosis. The total cost of surveillance during the 16-year period was nearly $2 400 000.

**Conclusion:**

Ultimately, serial imaging and the CA-125 assay detected the highest number of ovarian cancer and PCC progressive disease cases in comparison to physical examination and vaginal cytology, but nevertheless, all of the procedures were conducted at a considerable financial expense.

Ovarian cancer is the fifth most common cancer in women, accounting for nearly 15 280 deaths annually in the United States (Jemal *et al*, 2007). Standard treatment for ovarian carcinoma consists of surgery and adjuvant chemotherapy, to which ∼80% of patients will obtain a complete response (Fader and Rose, 2007; Gu *et al*, 2009). However, relapse rates are nearly 70% and the majority of patients eventually succumb to disease progression within 18–24 months (Gadducci *et al*, 1998, 2005; Berek *et al*, 1999; Fehm *et al*, 2005).

Primary peritoneal cancer (PPC) and ovarian cancer have similar phenotypic and histological characteristics (Eltabbakh *et al*, 1998). Nonetheless, as PPC has an undefined site of origin and the method of lymphatic dissemination is different from ovarian cancer, the disease may be a distinct, clinical entity. Moreover, studies have reported that PPC augurs a worse prognosis than ovarian cancer, particularly with regard to progression-free survival and overall survival (Eisenhauer *et al*, 2008).

The rationale for intensive surveillance of both ovarian and PPC is based on the premise that prompt detection and treatment of an asymptomatic patient will result in improved survival outcomes. Consequently, to detect recurrent cancer earlier and potentially improve upon patient prognosis, studies have attempted to evaluate follow-up protocol at the conclusion of primary treatment (Vaidya and Curtin, 2003; von Georgi *et al*, 2004).

Currently, there are only a limited number of formal, recognised guidelines for the surveillance of ovarian and PPC, particularly for previously treated patients who are without evidence of disease (U.S. Preventive Services Task Force, 1996; Vaidya and Curtin, 2003; von Georgi *et al*, 2004; Gadducci *et al*, 2007). The recommendations derived from the National Cancer Institute Consensus include evaluation of patient symptomatology, physical examination and monitoring of CA-125 levels every 3 months for the first 2 years following diagnosis (NIH, 1994). In patients for whom intensive screening is indicated, serial CT scans and CA-125 have been an integral component of follow-up and therapeutic management (Sugiyama *et al*, 1996; Fehm *et al*, 2005; Zola *et al*, 2007; Ferrandina *et al*, 2009). Conversely, physical examination and vaginal cytology have only offered limited predictive or survival benefits (Casey *et al*, 1996; Fehm *et al*, 2005).

Given the continued expense of obtaining serial gynaecologic cancer examinations, multiple serologic evaluations and imaging studies, one may conjecture that a comprehensive approach is both financially burdensome and anxiety laden for the patient (Wilson *et al*, 2004; Lee and Foster, 2009). Despite these concerns, there have been scant, large population studies addressing the cost and efficacy of conventional ovarian cancer screening measures (van Nagell *et al*, 2007; Havrilesky *et al*, 2008). In the present investigation, we documented the number and cost of evaluative procedures employed by a single gynaecologic oncology service over a 16-year period in the surveillance of initially diagnosed ovarian and PPCs. We hypothesise that the CA-125 and imaging will detect the highest number of recurrent ovarian and PPC cases in contrast to physical examination and vaginal cytology.

## Materials and methods

### Patients and eligibility criteria

This retrospective study involved all ovarian and PPC patients diagnosed and managed at a single, tertiary health care institution from November 1991 to February 2007. An institutional review board approved this retrospective study before any patient chart data were evaluated. Following an assessment of patient records from our clinical database, the charts of 1495 ovarian cancer patients were identified and reviewed. Only patients who were exclusively treated (e.g., underwent cytoreductive surgery and received all adjuvant therapy) and followed by the individual group of gynaecologic oncologists were eligible for this study. Patients who received neoadjuvant chemotherapy were excluded from the study evaluation.

### Surveillance protocol

Surveillance occurred at 3 month intervals for the first 2 years following diagnosis and completion of primary treatment, at 6-month intervals for the subsequent 3 years, and annually, thereafter. During each patient evaluation, a CA-125 assay was obtained and a physical examination was performed. CT scanning of the abdomen, chest and pelvis was ordered at 6-month intervals for the first 2 years of follow-up. Additional imaging (e.g., chest X-ray, pelvic ultrasound, PET scan) was ordered at the discretion of the physician. Vaginal cytology was obtained at the initial diagnosis and annually, thereafter.

Physical examination consisted of palpation of the abdomen, pelvis and lymph-node bearing areas. Abnormal vaginal cytology resulted in a subsequent colposcopic exam, and if indicated, a directed biopsy within 2 weeks. In the event of disease progression on CA-125 (>35 U ml^−1^), a repeat sample was taken within 4 weeks. If the CA-125 results remained positive for disease progression, but the findings on CT or physical exam were negative, a confirmatory CT scan and physical exam were scheduled. If any physical findings were present during the patient's exam, but the CA-125 and CT scan were negative, a follow-up on CA-125 was obtained within 4 weeks and a CT scan was ordered. If any of the previously described diagnostic measures identified disease progression >60 days following the initial detection, the patient data were excluded. We based this interval on the assumption that within this time frame, a medical intervention would already have occurred.

### Disease recurrence

Recurrent disease was defined as progression in surgically treated women with no evidence of disease following completion of primary therapy. If a patient's symptoms were noted during physical exam or history, but the CA-125, vaginal cytology and imaging results were negative, the initial recurrence was classified in accordance with the physical exam. If the patient exhibited disease progression on imaging, but had negative physical findings, a CA-125 within normal limits and normal vaginal cytology, the initial recurrence was categorised under imaging findings. If the patient had negative imaging, physical and vaginal cytology findings, but the CA-125 was indicative of disease progression, the initial recurrence was classified in accordance with the CA-125. When a patient presented with any combination of an elevated CA-125, physical findings or symptoms, abnormal vaginal cytology or positive imaging result, initial recurrence was categorised according to the diagnostic measure with the earliest recorded date.

In addition to the initial recurrence date, confirmatory dates for all positive diagnostic measures within a predetermined 60 days clinical time frame were documented for the purpose of comparative evaluation. Time to recurrence was defined as the period between initial diagnosis and documentation of disease recurrence. Recurrences were corroborated via histological, radiological or cytological evaluation. Once recurrent disease was established, individual surveillance data were concluded. Following a diagnosis of recurrent disease, all patients were offered additional treatment (e.g., surgery, radiation or chemotherapy). Overall survival was defined as time from the initial diagnosis until patient expiration, with all causes of death treated equally.

### Cost analysis

Cost analysis was based on previously published Southern California Medicare fee schedules for 2010. The combined (i.e., scan and radiologist evaluation) reimbursement rate for an abdomino-pelvic/chest CT scan (with and without contrast) was $1499.00. The combined reimbursement rate for a chest X-ray was $38.00. The combined reimbursements for a PET scan and pelvic ultrasound were $1414.00 and $144.00, respectively. The reimbursement for a physical examination was $140.00. The reimbursements for vaginal cytology and CA-125 were $47.00 and $87.00, respectively.

### Statistical analysis

All statistical analyses were conducted using MedCalc statistical software for biomedical research (version 9.5.1 for Windows; MedCalc Software, Maria Kerke, Belgium). The initial data analysis was conducted by employing a descriptive statistical approach, which underwent further examination via ANOVA. Significance (<0.05) was determined via two-sided *P*-values. Survival analyses were conducted via Kaplan–Meier.

## Results

From the original group of 1495 patients, 287 satisfied the established inclusionary criteria and comprised the study population. In this group, 240 had epithelial ovarian cancer (EOC), 23 had PPC and 24 had concurrent ovarian and uterine (COU) cancer. The median age of the entire study group was 59 years (range; 19–90). The clinicopathological characteristics for the study participants are exhibited in Tables 1 and 2.

In the study group, 151 patients developed disease progression. When examining the different detection modalities, CA-125 (*n*=74; 49.0% of cases) and imaging studies (*n*=66; 43.7%) detected the highest number of initial disease recurrence cases. Physical exam identified 10 (6.6%) cases. Vaginal cytology was the least effective measure, identifying only one (0.7%) recurrence.

**Figure 1:**
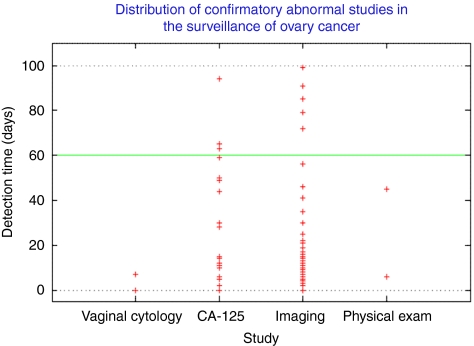
Distribution of confirmatory abnormal studies in the surveillance of ovary cancer.

A 60 days clinical interval was established to corroborate additional diagnoses with the initial abnormal surveillance test findings. Ultimately, the vast majority of diagnostic verifications occurred within this established time frame (Figure 1). Imaging studies confirmed recurrent disease in 45 patients. CA-125 corroborated disease recurrence in 20 patients. Physical exam and vaginal cytology were each able to confirm only two cases of recurrent cancer within the predetermined interval.

There were a total of 3569 imaging evaluations ordered over the 16-year study period. This resulted in a cost of $1 493 459 or 62.7% of the total surveillance cost. During the course of surveillance, 3560 physical exams were administered, resulting in a cost of $498 400. In terms of the CA-125, 4240 lab values were obtained; this resulted in a cost of $368 880. Finally, 457 vaginal cytology evaluations were obtained at a cost of $21 479.

**Figure 2:**
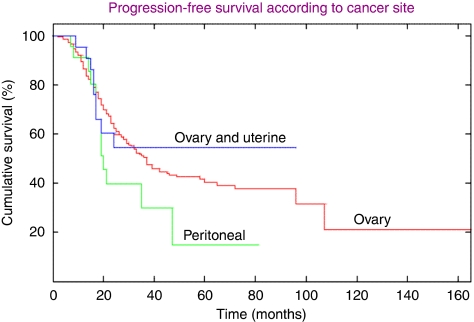
Progression-free survival according to cancer site.

An evaluation of cancer origin (EOC, PPC, COU) and progression-free interval (PFI) revealed significant differences (F(2284)=3.64; *P*=0.028). The EOC patients had a more favourable PFI (34.8 months) than the COU (24.8 months) and the PPC (22.7 months) patients (Figure 2). Disease stage (I/II-44.3 months *vs* III/IV-27.4 months) was also predictably associated with significant PFI differences (F(1,285)=33.09; *P*<0.001).

In all, 61 study patients have expired to date. Median OS has not been reached and current median length of patient follow-up is 42 months (range, 3–228).

## Discussion

Several studies have examined the utility of routine surveillance measures in ovarian cancer follow-up (Vaidya and Curtin, 2003; von Georgi *et al*, 2004; Gadducci *et al*, 2007), yet the issue remains a challenge for gynaecologic oncologists. Currently, follow-up procedures include serum CA-125 testing, physical examination, vaginal cytology and radiological imaging techniques.

In the present investigation, we examined recurrent disease detection rates for vaginal cytology, physical examination, CA-125 testing and CT imaging during a 16-year period at a single medical institution. Initially, we presumed that the CA-125 would have significant prognostic utility as previous reports have documented that increased levels coincided with disease progression in 56–94% of ovarian cancer patients (Rustin *et al*, 2001; Gadducci *et al*, 2004). We also contended that serial imaging would have high predictive value in detecting recurrent cancer (Funt *et al*, 2004; Gu *et al*, 2009).

In reviewing the CA-125 results, our evaluation revealed that serological evaluation identified the highest number (74 initial detections and 20 confirmatory diagnoses, 61% of all recurrences) of disease progression cases. Torizuka *et al* (2002) reported that the CA-125 was associated with an accuracy rate of 80% and a 100% positive predictive value in detecting recurrent ovarian cancer. García-Velloso *et al* (2007) reported a sensitivity of 57.6% and a specificity of 93.9% in the follow-up of platinum sensitive ovarian cancer patients. Conversely, Rustin and van der Burg (2009), in an EOC population, suggested that surveillance with serial CA-125 evaluation had limited impact on patient overall survival. Consequently, the CA-125 cannot be exclusively employed as a surveillance measure in ovarian cancer because elevated values may be ascertained in cases of non-malignancy (Rettenmaier *et al*, 2005). Further, although normalisation of CA-125 values may reflect disease regression, this condition is not assured. Ultimately, our results suggest that given the relatively low cost per patient recurrence ($3924) in the present investigation and ease of obtaining this measure, CA-125 evaluation appears beneficial in the surveillance of ovarian and PPC.

Imaging identified or confirmed 111 (72.1%) instances of disease recurrence; CT scanning encompassed the vast majority (77.9%) of radiological techniques within this category. However, overall imaging was extremely expensive (62.7% of the total surveillance cost throughout the study period); the cost for this procedure was $13 454 per recurrent cancer diagnosis. Sebastian *et al* (2008) retrospectively evaluated the diagnostic utility of CT scanning in 51 ovarian cancer patients with recurrent disease. They reported that CT of the chest and abdomen were associated with an accuracy rate of 89 and 79%, respectively. Conversely, studies have indicated that CT sensitivity may be compromised in ovarian cancer surveillance, particularly when there are retroperitoneal or lymphatic metastases (Coakley *et al*, 2002; Murakami *et al*, 2006). Despite the relatively high cost of ordering this diagnostic measure, CT potentially has promise in evaluating disease severity, thereby providing constructive insight into the determination of patient management (Funt *et al*, 2004).

Physical examination bestowed inadequate diagnostic utility in the current study, detecting only 6.7% of initial recurrences. Moreover, even when we included both initial and confirmatory diagnoses (12 cases), the procedure was associated with the highest cost ($41 533) per identified patient recurrence. In the Chan *et al* (2008) study, 41 (51%) of recurrent ovarian cancer patients presented with positive physical findings, of which only 3 were not corroborated by additional surveillance measures. Menczer *et al* (2006) reported similar findings; 78% of their recurrent ovarian cancer patients were diagnosed during a physical exam, but only 2 out of 43 progressive disease patients had exclusively, positive physical findings. Our results are in accordance with previous studies that suggest physical examination has only limited utility and financial viability in the long-term surveillance of ovarian cancer. Consequently, one could speculate that as ovarian cancer patients will continue to undergo a routine physical examination, the evaluation does not necessarily need to be conducted by a gynaecologic oncologist.

Vaginal cytology has been included in the intense follow-up of gynaecologic cancers (Santillan *et al*, 2008). However, this procedure has not proven to have sufficient external validity in effectuating a favourable clinical outcome (Milam *et al*, 2007). In the present investigation, vaginal cytology detected the least number of recurrent cases (one initial detection and two confirmatory), accounting for 2% of total cases. These results were similar to the 3.7% rate reported by Milam *et al* (2007), which indicated that the risk for ovarian cancer recurrence in the vaginal apex following hysterectomy was extremely low, even in advanced stage patients (Patsner *et al*, 1990; Milam *et al*, 2007).

In our analysis, the cost of obtaining vaginal cytology was $7159 per recurrent cancer diagnosis. Although this is significantly less than the $75 040 per patient recurrence reported by Bristow *et al* (2006) in their endometrial cancer study; our data, nevertheless, suggest that routine vaginal cytology does not have prognostic utility nor is it financially justifiable in ovarian cancer surveillance (Santillan *et al*, 2008).

Unsurprisingly, PPC patients exhibited the worst PFI (Eisenhauer *et al*, 2008). One may conjecture that their unfavourable prognosis was attributed to more extensive and voluminous disease (Aletti *et al*, 2009), although we did not further evaluate this premise. Conversely, we had suspected that the COU cancer patients might have exhibited a worse outcome, but essentially, the majority of the uterine tumours were small volume and low stage. Thus, the coexistence of two malignancies appeared to adversely affect patient prognosis only relative to the EOC patients (24.8 months *vs* 34.8). We recognise the complications associated with analysing the patients with concurrent ovarian and endometrial tumours because of the difficulty in determining which cancer actually recurred. However, as this cancer group comprised 10% of the patient population and clinicians will periodically encounter them, we contend that their surveillance data are of significant value.

In our long-term experience following patients with ovarian and PPCs, we ascertained that serial CA-125 determination and imaging appear more sensitive in detecting disease progression than physical examination and vaginal cytology. Therefore, in attempting to establish a more effective ovarian cancer surveillance programme, clinicians may consider altering the frequency of imaging and CA-125 evaluation based upon prognostic risk factors, such as disease stage and cancer origin, with intent to diagnose progressive disease earlier and potentially improve upon patient outcomes (Fehm *et al*, 2005). We do, however, recognise that in spite of significant research, ovarian cancer screening procedures have not proven to reduce patient mortality (Bast *et al*, 2007).

We appreciate that there are weaknesses inherent within this investigation. Study variability may be a concern as the evaluation was inadequately powered to discern any differences regarding approaches to surgery, pathological review and recommendations for adjuvant therapy. An analysis of patient symptomatology and progressive disease would have potentially benefited the investigation, but this data are often quite arbitrary and associated with poor predictive value (Rossing *et al*, 2010). Selection bias is another study concern, as the cohort represents a highly select group of patients who were all treated at a single institution. The final study results may also have been significantly biased because of the uneven distribution of the patient groups. In particular, as PPC patients have a very unfavourable prognosis, they should be evaluated independently.

We also appreciate that because the patients’ initial recurrence was categorised according to the diagnostic measure with the earliest recorded date, the results may have at least partially been based upon coincidence (i.e., the order in which the procedure was planned). In the present study, assessing the criteria according to the clinical scenario potentially confounds the comparative evaluation of the different surveillance tests.

Ultimately, as more than one surveillance method was clinically useful in detecting recurrent disease, a predetermined method (e.g., a biopsy) may have provided more adequate confirmatory data. This study also did not address the potential for significant lead-time variability between the recurrences identified via imaging or biology and the clinical manifestation of progressive disease. Therefore, we cannot comment specifically on whether the early detection of disease affects clinical outcome.

In regard to the cost of surveillance measures, we did not take into account any specific changes in the price of the various procedures. The study cost assessment would have also benefited from considering additional variables, such as lost productivity, travel time, and caregiver expenses related to obtaining these surveillance tests. Moreover, there is great difficulty in comparing the cost for procedures over an extended period of time. Finally, we began the investigation with 1495 patients, but only ended up with a sample size of 287 patients after applying the study exclusionary criteria. We may have been too stringent, but at the inception, the investigators wanted to ensure that all of the patient demographic, treatment and follow-up data were accessible and comprehensive. Despite the study limitations, we contend that the results from this extremely large study of ovarian and PPC patients are significant. Additional evaluation of routine surveillance in a larger, more diverse population of ovarian and PPC patients that incorporates economic costs should be strongly considered.

## Figures and Tables

**Table 1:** Ovarian and primary peritoneal cancer patient clinicopathological characteristics (*n*=287)

**Cancer origin**	***n* (%)**
Epithelial	240 (83.6)
Primary peritoneal	23 (8.0)
Concurrent ovarian and uterine	24 (8.4)
	
*Stage*	
IA	10 (3.5)
1B	2 (0.7)
IC	10 (3.5)
IIA	4 (1.4)
IIB	20 (7.0)
IIC	16 (5.6)
IIIA	6 (2.1)
IIIB	16 (5.6)
IIIC	181 (62.9)
IV	22 (7.7)
	
*Histology*	
Adenocarcinoma	22 (7.7)
Papillary serous	168 (58.5)
Endometrioid	55 (19.3)
Clear cell	29 (10.1)
Mucinous	10 (3.5)
Transitional	1 (0.3)
Adenosquamous	1 (0.3)
Psammocarcinoma	1 (0.3)
	
*Grade*	
G1	29 (10.1)
G2	58 (20.2)
G3	188 (65.5)
Unknown	12 (4.2)
Optimal surgery	255 (88.9)
Suboptimal surgery	32 (11.1)

**Table 2:** Pathological characteristics for the uterine component of the concurrent ovarian and uterine cancer patients (*n*=24)

**Stage**	***n* (%)**
IA	15 (62.5)
1B	3 (12.5)
IC	2 (8.3)
II	2 (8.3)
IIIC2	1 (4.2)
IVB	1 (4.2)
	
*Histology*	
Endometrioid	15 (62.5)
UPSC	4 (16.7)
Clear cell	3 (12.5)
Adenocarcinoma	2 (8.3)
	
*Grade*	
G1	6 (25.0)
G2	9 (41.7)
G3	8 (33.3)
	
*Lymphovascular space involvement*	
Yes	5 (20.8)
No	19 (79.2)

## References

[bib1] Aletti GD, Powless C, Bakkum-Gamez J, Wilson TO, Podratz KC, Cliby WA (2009) Pattern of retroperitoneal dissemination of primary peritoneum cancer: basis for rational use of lymphadenectomy. Gynecol Oncol 114: 32–36 19361840 10.1016/j.ygyno.2009.03.020

[bib2] Bast Jr RC, Brewer M, Zou C, Hernandez MA, Daley M, Ozols R, Lu K, Lu Z, Badgwell D, Mills GB, Skates S, Zhang Z, Chan D, Lokshin A, Yu Y (2007) Prevention and early detection of ovarian cancer: mission impossible? Recent Results Cancer Res 174: 91–100 17302189 10.1007/978-3-540-37696-5_9

[bib3] Berek JS, Trope C, Vergote I (1999) Surgery during chemotherapy and at relapse of ovarian cancer. Ann Oncol 10: 3–7 10.1023/a:1008338830718 10219446

[bib4] Bristow RE, Purinton SC, Santillan A, Diaz-Montes TP, Gardner GJ, Giuntoli II RL (2006) Cost-effectiveness of routine vaginal cytology for endometrial cancer surveillance. Gynecol Oncol 103: 709–713 16797686 10.1016/j.ygyno.2006.05.013

[bib5] Casey AC, Park M, Holschneider C, Bozuk M, Punyasavatsut M, Montz FJ (1996) Apical vaginal recurrence of ovarian carcinoma: presentation, treatment and survival. Int J Gynecol Cancer 6: 200–204

[bib6] Chan KK, Tam KF, Tse KY, Ngan HY (2008) The role of regular physical examination in the detection of ovarian cancer recurrence. Gynecol Oncol 110: 158–161 18544459 10.1016/j.ygyno.2008.04.030

[bib7] Coakley FV, Choi PH, Gougoutas CA, Pothuri B, Venkatraman E, Chi D, Bergman A, Hricak H (2002) Peritoneal metastases: detection with spiral CT in patients with ovarian cancer. Radiology 223: 495–499 11997559 10.1148/radiol.2232011081

[bib8] Eisenhauer EL, Sonoda Y, Levine DA, Abu-Rustum NR, Gemignani ML, Sabbatini PJ, Barakat RR, Chi DS (2008) Platinum resistance and impaired survival in patients with advanced primary peritoneal carcinoma: matched-case comparison with patients with epithelial ovarian carcinoma. Am J Obstet Gynecol 198: 213 e1–e7 18226627 10.1016/j.ajog.2007.07.003

[bib9] Eltabbakh GH, Piver MS, Natarajan N, Mettlin CJ (1998) Epidemiologic differences between women with extraovarian primary peritoneal carcinoma and women with epithelial ovarian cancer. Obstet Gynecol 91: 254–259 9469285 10.1016/s0029-7844(97)00650-9

[bib10] Fader AN, Rose PG (2007) Role of surgery in ovarian carcinoma. J Clin Oncol 25: 2873–2883 17617518 10.1200/JCO.2007.11.0932

[bib11] Fehm T, Heller F, Krämer S, Jäger W, Gebauer G (2005) Evaluation of CA125, physical and radiological findings in follow-up of ovarian cancer patients. Anticancer Res 25: 1551–1554 16033059

[bib12] Ferrandina G, Sallustio G, Fagotti A, Vizzielli G, Paglia A, Cucci E, Margariti A, Aquilani L, Garganese G, Scambia G (2009) Role of CT scan-based and clinical evaluation in the preoperative prediction of optimal cytoreduction in advanced ovarian cancer: a prospective trial. Br J Cancer 101: 1066–1073 19738608 10.1038/sj.bjc.6605292 PMC2768100

[bib13] Funt SA, Hricak H, Abu-Rustum N, Mazumdar M, Feldman H, Chi DS (2004) Role of CT in the management of recurrent ovarian cancer. AJR Am J Roentgenol 182: 393–398 14736669 10.2214/ajr.182.2.1820393

[bib14] Gadducci A, Cosio S, Carpi A, Nicolini A, Genazzani AR (2004) Serum tumor markers in the management of ovarian, endometrial and cervical cancer. Biomed Pharmacother 58: 24–38 14739059 10.1016/j.biopha.2003.11.003

[bib15] Gadducci A, Cosio S, Conte PF, Genazzani AR (2005) Consolidation and maintenance treatments for patients with advanced epithelial ovarian cancer in complete response after first-line chemotherapy: a review of the literature. Crit Rev Oncol Hematol 55: 153–166 15890524 10.1016/j.critrevonc.2005.03.003

[bib16] Gadducci A, Cosio S, Zola P, Landoni F, Maggino T, Sartori E (2007) Surveillance procedures for patients treated for epithelial ovarian cancer: a review of the literature. Int J Gynecol Cancer 17: 21–31 17291227 10.1111/j.1525-1438.2007.00826.x

[bib17] Gadducci A, Sartori E, Maggino T, Zola P, Landoni F, Fanucchi A, Palai N, Alessi C, Ferrero AM, Cosio S, Cristofani R (1998) Analysis of failures after negative second-look in patients with advanced ovarian cancer: an Italian multicenter study. Gynecol Oncol 68: 150–155 9514797 10.1006/gyno.1997.4890

[bib18] García-Velloso MJ, Jurado M, Ceamanos C, Aramendía JM, Garrastachu MP, López-García G, Richter JA (2007) Diagnostic accuracy of FDG PET in the follow-up of platinum-sensitive epithelial ovarian carcinoma. Eur J Nucl Med Mol Imaging 34: 1396–1405 17318549 10.1007/s00259-007-0366-9

[bib19] Gu P, Pan LL, Wu SQ, Sun L, Huang G (2009) CA 125, PET alone, PET-CT, CT and MRI in diagnosing recurrent ovarian carcinoma: a systematic review and meta-analysis. Eur J Radiol 71: 164–174 18378417 10.1016/j.ejrad.2008.02.019

[bib20] Havrilesky LJ, Sanders GD, Kulasingam S, Myers ER (2008) Reducing ovarian cancer mortality through screening: is it possible, and can we afford it? Gynecol Oncol 111: 179–187 18722004 10.1016/j.ygyno.2008.07.006

[bib21] Jemal A, Siegel R, Ward E, Murray T, Xu J, Thun MJ (2007) Cancer statistics, 2007. CA Cancer J Clin 57: 43–66 17237035 10.3322/canjclin.57.1.43

[bib22] Lee DW, Foster DA (2009) The association between hospital outcomes and diagnostic imaging: early findings. J Am Coll Radiol 6: 780–785 19878885 10.1016/j.jacr.2009.08.007

[bib23] Menczer J, Chetrit A, Sadetzki S, Golan A, Levy T (2006) Follow-up of ovarian and primary peritoneal carcinoma: the value of physical examination in patients with pretreatment elevated CA125 levels. Gynecol Oncol 103: 137–140 16564077 10.1016/j.ygyno.2006.02.005

[bib24] Milam MR, Sood AK, King S, Bassett Jr RL, Lu KH, Slomovitz BM, Coleman RL, Ramirez PT (2007) Supracervical hysterectomy in patients with advanced epithelial ovarian cancer. Obstet Gynecol 109: 641–646 17329515 10.1097/01.AOG.0000257117.78230.0f

[bib25] Murakami M, Miyamoto T, Iida T, Tsukada H, Watanabe M, Shida M, Maeda H, Nasu S, Yasuda S, Yasuda M, Ide M (2006) Whole-body positron emission tomography and tumor marker CA125 for detection of recurrence in epithelial ovarian cancer. Int J Gynecol Cancer 16: 99–107 16515575 10.1111/j.1525-1438.2006.00471.x

[bib26] NIH (1994) Ovarian cancer: screening, treatment, and follow-up. NIH Consensus Statement 12: 1–30 7881476

[bib27] Patsner B, Orr Jr JW, Mann Jr WJ, Taylor PT, Partridge E, Allmen T (1990) Does serum CA-125 level prior to second-look laparotomy for invasive ovarian adenocarcinoma predict size of residual disease? Gynecol Oncol 38: 373–376 2227551 10.1016/0090-8258(90)90076-w

[bib28] Rettenmaier MA, Goldstein BH, Stallman JM, Brown III JV, Micha JP (2005) Greatly elevated serum CA-125 level in a patient with a ruptured endometrioma. J Gynecol Surg 21: 109–112

[bib29] Rossing MA, Wicklund KG, Cushing-Haugen KL, Weiss NS (2010) Predictive value of symptoms for early detection of ovarian cancer. J Natl Cancer Inst 102: 222–229 20110551 10.1093/jnci/djp500 PMC2826180

[bib30] Rustin GJ, Marples M, Nelstrop AE, Mahmoudi M, Meyer T (2001) Use of CA-125 to define progression of ovarian cancer in patients with persistently elevated levels. J Clin Oncol 19: 4054–4057 11600607 10.1200/JCO.2001.19.20.4054

[bib31] Rustin GJ, van der Burg ME (2009) A randomized trial in ovarian cancer (OC) of early treatment of relapse based on CA125 level alone versus delayed treatment based on conventional clinical indicators. J Clin Oncol 27: 18s

[bib32] Santillan A, Govan L, Zahurak ML, Diaz-Montes TP, Giuntoli II RL, Bristow RE (2008) Feasibility and economic impact of a clinical pathway for pap test utilization in Gynecologic Oncology practice. Gynecol Oncol 109: 388–393 18405946 10.1016/j.ygyno.2008.01.006

[bib33] Sebastian S, Lee SI, Horowitz NS, Scott JA, Fischman AJ, Simeone JF, Fuller AF, Hahn PF (2008) PET-CT vs. CT alone in ovarian cancer recurrence. Abdom Imaging 33: 112–118 17404789 10.1007/s00261-007-9218-0

[bib34] Sugiyama T, Nishida T, Komai K, Nishimura H, Yakushiji M, Nishimura H (1996) Comparison of CA 125 assays with abdominopelvic computed tomography and transvaginal ultrasound in monitoring of ovarian cancer. Int J Gynaecol Obstet 54: 251–256 8889633 10.1016/0020-7292(96)02721-x

[bib35] Torizuka T, Nobezawa S, Kanno T, Futatsubashi M, Yoshikawa E, Okada H, Takekuma M, Maeda M, Ouchi Y (2002) Ovarian cancer recurrence: role of whole-body positron emission tomography using 2-[fluorine-18]-fluoro-2-deoxy- D-glucose. Eur J Nucl Med Mol Imaging 29: 797–803 12029554 10.1007/s00259-001-0750-9

[bib36] U.S. Preventive Services Task Force (1996) Guide to Clinical Preventive Services (2nd edn). National Institute of Health: Washington, DC

[bib37] Vaidya AP, Curtin JP (2003) The follow-up of ovarian cancer. Semin Oncol 30: 401–412 12870142 10.1016/s0093-7754(03)00100-3

[bib38] van Nagell Jr JR, DePriest PD, Ueland FR, DeSimone CP, Cooper AL, McDonald JM, Pavlik EJ, Kryscio RJ (2007) Ovarian cancer screening with annual transvaginal sonography: findings of 25,000 women screened. Cancer 109: 1887–1896 17373668 10.1002/cncr.22594

[bib39] von Georgi R, Schubert K, Grant P, Münstedt K (2004) Post-therapy surveillance and after-care in ovarian cancer. Eur J Obstet Gynecol Reprod Biol 114: 228–233 15140520 10.1016/j.ejogrb.2003.10.029

[bib40] Wilson CM, Tobin S, Young RC (2004) The exploding worldwide cancer burden: the impact of cancer on women. Int J Gynecol Cancer 14: 1–11 10.1111/j.1048-891x.2004.14178.x 14764024

[bib41] Zola P, Fuso L, Mazzola S, Gadducci A, Landoni F, Maggino T, Sartori E (2007) Follow-up strategies in gynecological oncology: searching appropriateness. Int J Gynecol Cancer 17: 1186–1193 17466042 10.1111/j.1525-1438.2007.00943.x

